# Mechanisms of action and advances in application of music therapy in improving cognitive impairment based on the theory of neuroplasticity

**DOI:** 10.3389/fnins.2026.1828930

**Published:** 2026-05-28

**Authors:** Zi-Tong Zeng, Jia-Xin Tang, Si-Rong Wang, Li-Zhu Lei, Zi-Yi Xing, Jian-Mei Wang, Jing Wu

**Affiliations:** 1 School of Integrated Traditional Chinese and Western Medicine, Hunan University of Chinese Medicine, Hunan, China; 2 School of Traditional Chinese Medicine, Hunan University of Chinese Medicine, Hunan, China; 3 School of Medicine, Hunan University of Chinese Medicine, Hunan, China; 4 School of Sport Arts, Hunan University of Chinese Medicine, Hunan, China

**Keywords:** clinical evidence, cognitive impairment, neural resonance mechanism, neurologic music therapy, neuroplasticity

## Abstract

As global population aging accelerates, preventing and treating cognitive impairment has become a major public health priority. Music therapy is a safe, well-tolerated non-pharmacological intervention with substantial potential to improve cognitive function. This review synthesizes the neurologic music therapy (NMT) framework, encompassing techniques targeting attention, memory, and executive function, delivered through both active and receptive approaches. Clinical investigations indicate that music therapy may improve cognition and neuropsychiatric symptoms in Alzheimer’s disease (AD), vascular cognitive impairment (VCI), Parkinson’s disease–related cognitive impairment, mild cognitive impairment (MCI), and traumatic brain injury (TBI); however, effects appear to vary by intervention duration and disease stage. This narrative review aims to provide a theoretical foundation and practical guidance for the non-pharmacological intervention of cognitive impairment by collating evidence on the neuroplasticity theoretical foundations, technical systems, and clinical applications of music therapy.

## 1 Introduction

Cognitive impairment refers to acquired deficits in cognitive domains such as memory, executive function, and language, and encompasses a continuum ranging from MCI to dementia (Sachdev et al., 2014; Morozova et al., 2022). Globally, approximately 15% of older adults have MCI (Bai et al., 2022). In China, the prevalence is 15.5% among individuals aged 60 and above, affecting an estimated 38.77 million people (Jia et al., 2020). More concerningly, 10–15% of individuals with MCI progress to dementia each year, a rate substantially higher than the 1–2% observed in the general population (McGrattan et al., 2022). Although drugs such as cholinesterase inhibitors are widely used to treat AD, their efficacy is limited, they are associated with significant adverse effects, and they provide little improvement in neuropsychiatric symptoms (Moreta et al., 2021). Therefore, NMT, a non-pharmacological intervention that has garnered considerable attention because it induces neuroplastic changes and provides multimodal stimulation (Chatterjee et al., 2021), has been increasingly applied in the rehabilitation of cognitive, sensory, and motor dysfunctions arising from neurological disorders (Moschonas et al., 2023; Wei and Qiao, 2024). This narrative review evaluates how music-based interventions may enhance the frontoparietal executive control network via rhythmic entrainment and facilitate autobiographical memory retrieval by engaging the hippocampal–amygdala circuit through emotion–memory coupling, thereby modulating attention, memory, and executive-function domains to improve cognition in individuals with specific neurological disorders.

## 2 The neuroplasticity theoretical basis of music therapy

### 2.1 Multilayered mechanisms of music-induced neural plasticity

Music-induced neural plasticity refers to the adaptive remodeling of brain structure and function driven by musical activities through multisensory stimulation (Schlaug, 2015). Wang J. et al. (2023) exposed juvenile mice to music with different tones and found that musical stimulation upregulated the expression of brain-derived neurotrophic factor (BDNF) and its receptor, tropomyosin receptor kinase B (TrkB), thereby activating downstream signaling pathways, including phospholipase Cγ1 (PLCγ1)–protein kinase C (PKC), phosphatidylinositol 3-kinase (PI3K)–protein kinase B (AKT), and mitogen-activated protein kinase (MAPK)–extracellular signal-regulated kinase (ERK), and promoting dendritic spine formation and synaptic protein expression in the hippocampus and prefrontal cortex. Clinical studies in patients with AD have reported that music-based interventions can improve cognitive function. These improvements may be associated with the molecular mechanisms described above. However, direct causal evidence from clinical trials is currently lacking (Gómez Gallego and Gómez García, 2017). Cheng et al. (2024) further confirmed in a mouse model of depression induced by chronic unpredictable mild stress that music intervention enhances hippocampal neurogenesis and structural plasticity by increasing the number of DCX-positive newborn neurons in the dentate gyrus. At the brain network level, Slade et al. (2025) hypothesized based on a narrative review that music training enhances the anterior cingulate cortex-pre-supplementary motor area (ACC-pre-SMA) node within the salience network (SN). This enhancement strengthens its driving effect on the frontoparietal control network (FPCN), thereby improving executive control efficiency. However, the model currently lacks direct experimental evidence from animal studies, clinical trials, or neuroimaging.

### 2.2 Neural networks for music processing and resonance mechanisms

**FIGURE 1:**
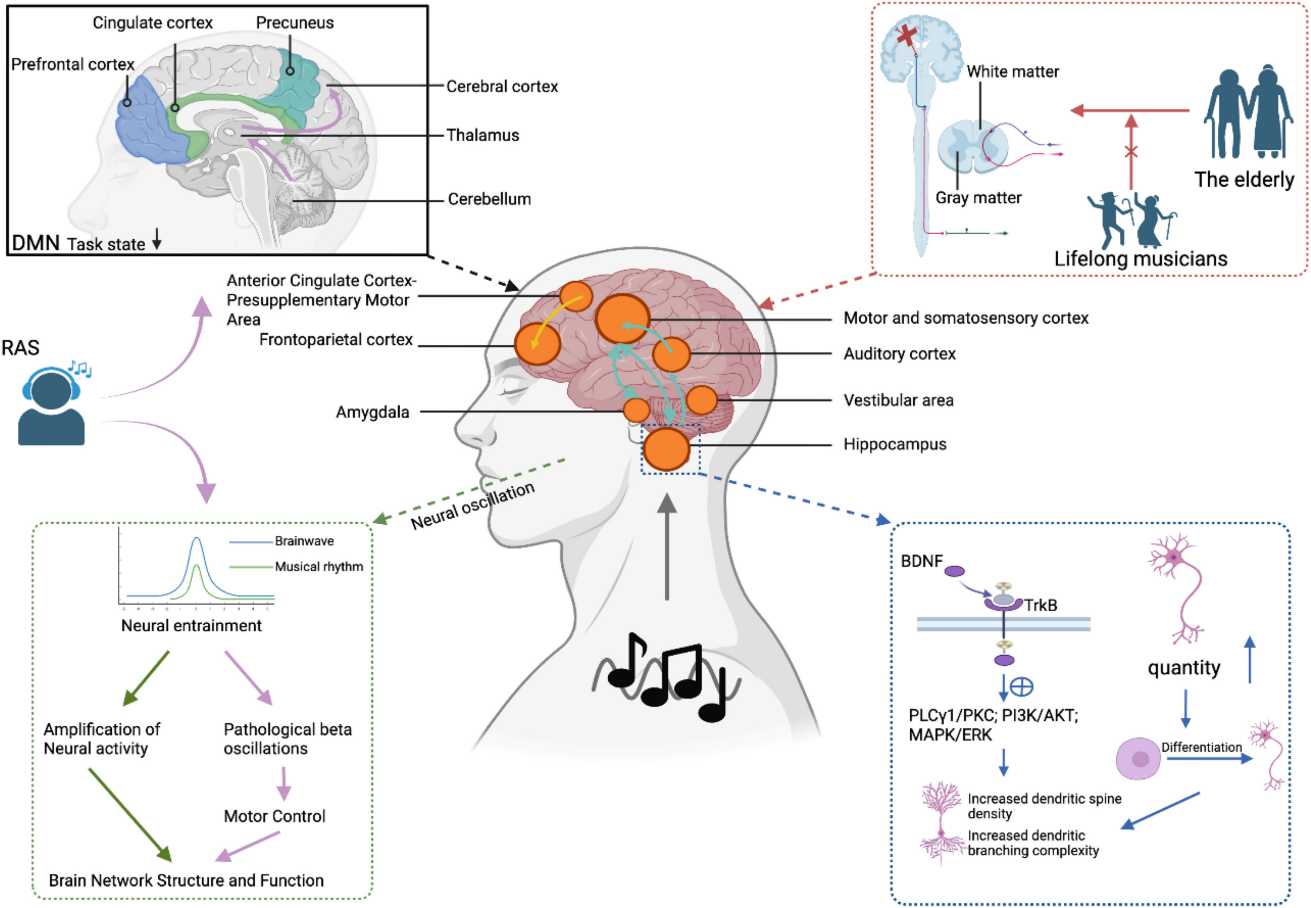
Multi-level mechanisms of music therapy-induced neuroplasticity in cognitive function improvement. Main panel: Lateral view of the brain highlighting activated regions, including the motor and somatosensory cortices, auditory cortex, vestibular area, hippocampus, amygdala, frontoparietal cortex, and anterior cingulate cortex/pre-supplementary motor area. Insets: (top left) During task states, DMN shows reduced connectivity (prefrontal and cingulate cortices, precuneus); RAS engages the cerebello-thalamo-cortical circuit, which may support cognitive improvement. (Left) RAS entrainment: rhythmic cues synchronize neural oscillations, increase task-relevant neural activity, suppress pathological beta-band oscillations, and enhance motor control. (Top right) Lifelong musicians show better preservation of age-related white-matter integrity in the bilateral superior longitudinal fasciculus and uncinate fasciculus than non-musicians, consistent with cognitive reserve. (Bottom right) BDNF–TrkB signaling pathways (PLCγ1/PKC, PI3K/AKT, and MAPK/ERK) promote dendritic spine formation, increased dendritic branching complexity, and neuronal differentiation. BDNF, Brain-Derived Neurotrophic Factor; TrkB, Tropomyosin Receptor Kinase B; PLCγ1, Phospholipase C Gamma 1; PKC, Protein Kinase C; PI3K, Phosphatidylinositol 3-Kinase; AKT, Protein Kinase B; MAPK, Mitogen-Activated Protein Kinase; ERK, Extracellular Signal-Regulated Kinase; RAS, Rhythmic Auditory Stimulation. Figure was created with BioRender.com under paid license.

Music therapy induces functional coupling across multiple brain regions through neural resonance mechanisms. By immediately modulating emotion and motor control, this process further drives structural and functional reorganization of brain networks, thereby improving cognitive function. Its mechanistic framework constructed based on the evidence in this manuscript is illustrated in Figure 1. Music-induced emotional processing relies on the functional coupling of various brain areas. The auditory cortex and premotor cortex are involved in cross-vocal-timbre emotion categorization (Paquette et al., 2018), whereas the motor and somatosensory cortices are involved in rhythmic synchronization and sensorimotor coupling (Liu et al., 2018; Guo et al., 2023). Limbic structures, such as the amygdala and hippocampus, are dedicated to processing specific emotions (Putkinen et al., 2021). The neural resonance mechanism is a key target of music therapy techniques such as rhythmic auditory stimulation (RAS), in which neural activity is selectively enhanced when external rhythms align with the brain’s endogenous oscillatory frequencies (e.g., alpha and theta waves) (Sreeraj et al., 2019). Koshimori and Thaut (2023) demonstrated that RAS, acting as an exogenous pacemaker, can modulate pathological beta oscillations via neural entrainment, thereby enhancing motor cortex modulation and frontoparietal cross-frequency coupling, which in turn improves motor control. Wu et al. (2022) demonstrated through clinical imaging experiments that RAS can also activate the cerebello-thalamo-cortical pathway to establish compensatory mechanisms, thereby compensating for functional brain damage in individuals with cognitive impairments. Long-term neural resonance can reshape brain network structure and function. Structural connectivity, which is based on white matter integrity, serves as the material basis for functional connectivity. In older adults, white matter fiber disruption and volume reduction lead to weakened functional connectivity (Basile et al., 2022; Merenstein et al., 2025). Specifically, reduced within-network connectivity accompanied by enhanced between-network connectivity, decreased connectivity among task-related brain regions, and a diminished negative correlation between the task-positive network and the DMN collectively contribute to cognitive decline (Xia et al., 2022). Andrews et al. (2023), using diffusion tensor imaging, found that lifelong musicians exhibit a positive correlation between age and white matter integrity in the bilateral superior longitudinal fasciculus and uncinate fasciculus, whereas non-musicians show the typical negative correlation. However, the small sample size and cross-sectional design of this study limit causal inference; thus, the findings only suggest that lifelong musical training may alter the trajectory of age-related white-matter degeneration and confer cognitive reserve. Tewarie et al. (2019) reported that functional connectivity is contingent on dynamic coupling formed by neural oscillations. Bleibel et al. (2023) demonstrated that music therapy can promote functional recovery and emotional regulation by enhancing neural plasticity.

## 3 The neurologic music therapy system and mechanisms of action

### 3.1 Classification of techniques based on cognitive domains

Within the NMT framework, interventions can be classified by cognitive domains and modes of engagement into two types: standardized intervention protocols and descriptive classification Music Attention Control Training (MACT), Associative Mood and Memory Training (AMMT), and Musical Executive Function Training (MEFT), all classified by cognitive domain, originate from the standardized registered protocols (marked with ^®^) presented in the *Handbook of Neurologic Music Therapy* (Hakvoort et al., 2025, De L’Etoile and Jones, 2025, Hegde et al., 2025). These protocols possess well-defined technical specifications and scopes of application By contrast, Active Music Intervention (AMI) and Receptive Music Intervention (RMI), based on a descriptive categorization of engagement modes, correspond to interventions involving performance and singing, and to listening-based interventions, respectively, and exhibit high internal operational heterogeneity. RAS is also one of the standard techniques defined in the handbook and frequently appears as a core module of AMI (Thaut and Rice, 2025). The following provides a point-by-point introduction and explanation.

#### 3.1.1 Music Attention control training

MACT is a core intervention technique in NMT that specifically targets cognitive dysfunction. By designing structured musical tasks, this technique systematically uses musical elements—such as rhythm, melody, and harmony—to target and train subcomponents of attention, including focused, selective, sustained, and alternating attention (Hakvoort et al., 2025). Its theoretical foundation is the three-network model of attention proposed by Posner and colleagues, comprising the alerting network (arousal and maintenance of a vigilant state), the orienting network (information filtering and orienting), and the executive control network (conflict monitoring and cognitive control) (de Souza Almeida et al., 2021). Based on this neurotheoretical framework, the clinical efficacy of MACT has been supported by evidence-based studies across diverse clinical populations, including individuals with acquired brain injury (Jones et al., 2021; Shin and Jeong, 2023), schizophrenia (van Alphen et al., 2019), and neurodevelopmental disorders in children and adolescents, such as attention-deficit/hyperactivity disorder (Abrahams and van Dooren, 2018) and autism spectrum disorder (Pasiali et al., 2014). The neurobiological mechanisms of this training method operate at multiple levels (see Table 1), mainly including the following:

**TABLE 1:** Neural mechanisms of MACT.

Target brain regions	Neural mechanisms	Attentional function	References
Prefrontal-parietal network	Neural oscillatory entrainment.	Prioritizes task-relevant information and suppresses distractors, thereby enhancing selective attention.	(Tallon-Baudry, 2012; Harding et al., 2025)
Brainstem, ascending reticular activating system	Brainstem auditory reflex pathways.	Rapidly activates alerting networks, facilitating rapid mobilization and optimal allocation of attentional resources.	(Juslin and Västfjäll, 2008)
Parietal cortex, anterior cingulate cortex, and basal ganglia network	Functional synergy across multiple regions and network integration.	Synergistically enhances executive control and attentional regulation.	(Vuust et al., 2022)

#### 3.1.2 Associative mood and memory training

AMMT is a specialized intervention technique within NMT designed to address impairments in memory function. This technique is based on neural coupling between emotion and memory. It involves the careful selection and presentation of personally familiar music that elicits strong emotional responses, thereby facilitating autobiographical memory retrieval (De L’Etoile and Jones, 2025). Autobiographical memory, which integrates episodic and semantic memory systems, plays a crucial role in maintaining an individual’s self-identity (Compère et al., 2021; Ranganath, 2022). AMMT is primarily applicable to patients with neurodegenerative memory disorders, such as AD and VD, as well as to individuals experiencing memory deficits following brain injury or stroke (De L’Etoile and Jones, 2025). A detailed description of its multilevel neural mechanisms is provided in Table 2.

**TABLE 2:** Neural mechanisms of AMMT.

Target brain regions	Neural mechanisms	Memory function	References
Limbic system (amygdalo-hippocampal circuit)	Uses music-induced emotion as a “retrieval key” to engage emotion-memory coupling circuits.	Enhances the efficiency of autobiographical memory retrieval.	(Frühholz et al., 2014; Kensinger and Ford, 2020)
Music processing–related brain regions (caudal anterior cingulate cortex, ventral supplementary motor area)	Leverages relatively preserved musical nodes in pathological states as bridges to engage associated memory networks.	Promotes the mobilization of holistic episodic memory.	(Jacobsen et al., 2015; Castro et al., 2020)
Neural network connectivity (frontotemporal lobes, cortico-cerebellar connections)	Repetitive training induces bidirectional plasticity: it may optimize network efficiency by reducing redundant connections at the MCI stage and may elicit compensatory mechanisms by enhancing functional connectivity at the AD stage.	Dynamic adaptation mechanisms of “streamlining” and “enhancement” support memory.	(King et al., 2019; Fischer et al., 2021)

#### 3.1.3 Musical executive function training

MEFT systematically enhances executive functions through active music-creation activities (Hegde et al., 2025). Executive functions encompass three core components—working memory, cognitive flexibility, and inhibitory control—as well as higher-order functions such as planning, decision-making, and problem-solving (Bombonato et al., 2024; Hegde et al., 2025). MEFT is applicable to patients with neurological disorders (e.g., brain injury, attention-deficit/hyperactivity disorder, and PD) and patients with neuropsychiatric disorders (e.g., depression and schizophrenia), as well as healthy older adults, individuals with MCI, and healthy individuals seeking functional optimization (Hegde et al., 2025). The neural mechanisms underlying MEFT are detailed in Table 3.

**TABLE 3:** Neural mechanisms of MEFT.

Target brain regions	Neural mechanisms	Executive function	References
Dorsolateral prefrontal cortex	Generates and maintains internal representations and sustained neuronal firing.	Working memory	(Chafee and Heilbronner, 2022)
Lateral prefrontal cortex	Anteriorly, the ventral pathway couples with the temporal lobe; posteriorly, the dorsal pathway interacts with the parieto-motor cortex network.	Musical structure planning; motor planning and hierarchical action control, from abstract structural rules to specific motor execution.	(Bianco et al., 2021)
Anterior cingulate cortex	Continuously monitors conflicts arising during music creation and communicates them to the prefrontal cortex.	Cognitive control	(Kerns et al., 2004; de Aquino et al., 2019)
Basal Ganglia	The striatal direct pathway (D1-MSNs) disinhibits target actions, whereas the indirect pathway (D2-MSNs) increases inhibition of competing actions.	Action selection	(Friend and Kravitz, 2014)
	Facilitates the shift from discrete notes to fluid phrases, enabling a shift from static planning to dynamic sequencing.	Sequence learning	(Shi and Zhang, 2020)

### 3.2 Classification of intervention models based on participation format

#### 3.2.1 Active music intervention

AMI integrates auditory feedback, motor planning, and attentional control through activities such as instrument playing, singing, and rhythmic training, thereby promoting multisensory synergistic processing (Gómez-Gallego et al., 2021; Schneider et al., 2022; Kunikullaya et al., 2025). The underlying neural mechanisms involve precise coordination between auditory and motor systems. Segado et al. (2021) reported that instrumental performance and singing activate a broad network of brain regions, including the primary auditory cortex, posterior superior temporal gyrus, intraparietal sulcus, supramarginal gyrus, dorsal premotor cortex, and primary motor cortex. Furthermore, Herrojo Ruiz et al. (2017) reported that auditory–motor sequence learning is synchronized with beta oscillations (13–30 Hz) in the cingulate gyrus and cerebellum, thereby mediating temporal coupling between sensory and motor processes.

RAS, a core technique within AMI (Schneider et al., 2022), leverages neural entrainment between regular beats and the brain’s intrinsic oscillations to improve temporal processing and motor-cognitive functions (Lakatos et al., 2008; Emmery et al., 2023). Building on the Auditory Predictive Motor Simulation hypothesis, Cannon and Patel (2021) proposed that beat perception modulates neural oscillations and recruits motor-planning regions for predictive simulation, and that RAS reinforces this process through active synchronization. Bella et al. (2017) reported that RAS can improve gait parameters in patients with PD. Additionally, Emmery et al. (2023) reported that RAS may promote post-stroke motor rehabilitation and cognitive recovery in MCI.

#### 3.2.2 Receptive music intervention

RMI, which involves listening to self-selected music to facilitate emotional expression, offers unique advantages in ameliorating psychological symptoms such as anxiety and depression (Tsoi et al., 2018). Its effects are mediated by activation of the reward system, involving regions such as the striatum, dopaminergic midbrain nuclei, amygdala, insula, prefrontal cortex, and orbitofrontal cortex (Zatorre, 2015). Using positron emission tomography, Salimpoor et al. (2011) demonstrated that music-evoked emotional peaks are accompanied by dopamine release in the striatum. The caudate nucleus and the nucleus accumbens are activated during the anticipatory phase and peak experience, respectively, reflecting the temporal dynamics of this process.

Furthermore, music listening can modulate neuroendocrine function via the autonomic nervous system and the hypothalamic–pituitary–adrenal axis, thereby mitigating stress responses (Lata and Kourtesis, 2021; Raglio, 2023). Mojtabavi et al. (2020) reported that listening to relaxing music enhances parasympathetic activity, increases the high-frequency component of heart rate variability, and reduces heart rate and respiratory rate. Zhang et al. (2020) demonstrated that therapist-selected music—characterized by a broader audio frequency range, a more structured musical form, and greater cultural appropriateness—more effectively activates the autonomic nervous system in patients with disorders of consciousness than patient-preferred music. This superiority is particularly evident in indices of sympathetic activation, overall ANS activity, and thermoregulatory stability. Fast-tempo music lowers cortisol levels, whereas slow-tempo music increases oxytocin levels and promotes physiological relaxation (Ooishi et al., 2017), thereby reducing subjective anxiety and inducing positive emotions (Lata and Kourtesis, 2021).

## 4 Music therapy based on the theory of neuroplasticity in improving cognitive impairment

The intervention protocols of the NMT technical system, such as MACT, AMMT, MEFT, and RAS, although targeting distinct cognitive domains including attention, memory, and executive functions, all have therapeutic pathways that rely on neural processing mediated via the auditory pathway. AMMT leverages melodic and harmonic structures to induce activation of the emotional memory circuit and enhance functional connectivity within the hippocampal-amygdala complex (Frühholz et al., 2014; Kensinger and Ford, 2020). RAS, through neural entrainment mechanisms, achieves the phase-locking of exogenous rhythms with endogenous θ/α oscillations, thereby driving the prefrontal-parietal executive control network (Lakatos et al., 2008; Emmery et al., 2023). This section organizes the clinical application evidence of neuro-music therapy in patients with cognitive impairment such as AD, VCI, and PD.

### 4.1 Alzheimer’s disease

Alzheimer’s disease (AD) is a neurodegenerative disorder characterized by progressive cognitive decline (Scheltens et al., 2021) and behavioral and psychological symptoms of dementia (BPSD) (Calsolaro et al., 2021). Music therapy, as a non-pharmacological intervention, has shown efficacy in improving cognitive function and BPSD in patients with AD (Bleibel et al., 2023; Wang S. G. et al., 2023; Zhou et al., 2025). Table 4 summarizes progress in the specific applications of music therapy. Notably, in individuals with mild AD, improvements following music interventions are primarily observed in global cognition and memory, whereas in those with moderate-to-severe AD, benefits are mainly reflected in BPSD. Whether the differential therapeutic effects that emerge with disease progression are attributable to changes in neuroplasticity targets, or, in moderate-to-severe stages, to the limited sensitivity of cognitive assessment tools and the practical difficulties in implementing active-participation paradigms, warrants further verification.

**TABLE 4:** Advances in the application of music therapy in improving cognitive impairment in AD patients.

Disease		Intervention description	Intervention duration	Clinical assessment measures			Efficacy	Mechanism of action	References
AD	Mild	Listening to emotionally evocative music	5 weeks	Baseline MMSE (mean)	14.6	Cognitive function	Improved autobiographical memory retrieval.	Induces emotional states and enhances attention and arousal.	(Meilán García et al., 2012)
		Listening to classical music (Mozart, Pachelbel)	6 months		17.7 ± 5.98		Significantly improved abstraction (*p* = 0.024).	Activates prefrontal and temporal cortical circuits to enhance neuroplasticity.	(Li et al., 2015)
	Mild to moderate	AMI: welcome songs, body movement; RMI: listening to songs.	6 weeks		15.02 ± 5.40		Significantly Improved memory (*p* = 0.000, η^2^ = 0.69), orientatio (*p* = 0.000, η^2^ = 0.76), and language abilities (*p* = 0.047, η^2^ = 0.27).	Enhances cognitive efficiency via emotion-memory pathways, promoting brain network reorganization and right-hemisphere language compensation.	(Gómez Gallego and Gómez García, 2017)
		AMI: singing, rhythmic exercises, dancing; RMI: seated listening to personalized music	3 months		AMI group: 17.79 ± 3.90; RMI group: 18.28 ± 6.14		AMI significantly improved MMSE scores (*p* < 0.001, η^2^ = 0.62).	Activates multi-brain-region networks, promotes neuroplasticity, and enhances memory and executive functions.	(Gómez-Gallego et al., 2021)
	Mild	Listening to classical music (Mozart, Pachelbel)	6 months	Baseline NPI (mean)	7.4 ± 13.17	BPSD	There was no significant improvement in BPSD (*p* = 0.431), but MT group showed numerically less deterioration.	Modulates limbic system and parasympathetic activity, stabilizes emotional states, and alleviates behavioral and psychological symptoms.	(Li et al., 2015)
	Mild to moderate	AMI: welcome songs, body movement; RMI: listening to songs	6 months		17.71 ± 11.59		Alleviated BPSD, including anxiety (*p* = 0.002, η^2^ = 0.53), delusions (*p* = 0.015, η^2^ = 0.37), hallucinations (*p* = 0.026,η^2^ = 0.33), agitation (*p* = 0.010, η^2^ = 0.41), and irritability (*p* = 0.025, η^2^ = 0.33).	Reduces physiological stress responses, evokes positive emotions, and promotes social interaction.	(Gómez Gallego and Gómez García, 2017)
		AMI: singing, rhythmic exercises, dancing; RMI: seated listening to personalized music	3 months		AMI group: 20.92 ± 9.20; RMI group: 18.38 ± 7.48		AMI significantly reduced NPI scores (η^2^ = 0.61, *p* < 0.001).	Regulates the stress system and emotional responses and improves behavioral symptoms.	(Gómez-Gallego et al., 2021)
		Group music intervention, percussion instrument playing	12 weeks		/		Anxiety improved significantly (*P* < 0.001), whereas depressive symptoms showed no significant improvement (*P* = 0.214).	Evokes emotional memories, enhances interpersonal interaction, and increases alertness through instrumental touch.	(Liu et al., 2020)
	Moderate to severe	Group music therapy combined with AMI and RMI	6 weeks	Baseline BEHAVE-AD (mean)	5.5		The treatment group showed significant improvement in activity disorders (*p* = 0.02) and a significant reduction in aggressive and anxious symptoms (*p* < 0.01).	Alleviates behavioral and psychological symptoms triggered by factors such as difficulties in environmental recognition.	(Svansdottir and Snaedal, 2006)
	Severe	AMI: listening to personalized music; RMI: clapping, singing, dancing	10 weeks		Paranoia/delusions: 2.1–2.2; Movement disorders: 2.3–3.0		In alleviating BPSD (e.g., emotional distress, anxiety, paranoia, and behavioral disturbances), AMI shows superior efficacy to RMI.	Enhances parasympathetic activity and evokes positive emotions.	(Sakamoto et al., 2013)
	Mild, moderate, severe	AMI: singing RMI: listening to music	3 months	Baseline MMSE (mean)	13.45 ± 3.66	Cognitive function	Significantly improved immediate recall, delayed recall, and verbal fluency in mild AD (*p* < 0.05), with no significant effect in moderate-to-severe AD.	Activates retained cortical and subcortical brain regions.	(Lyu et al., 2018)
				Baseline NPI (mean)	26.18 ± 13.25	BPSD	Significant improvement in BPSD for severe AD and in caregiver burden for moderate-to-severe AD, with no significant effect in mild AD.	Engages brain regions regulating emotion and behavior, together with the positive social and emotional experiences inherent in singing.	

MMSE: Mini-Mental State Examination (Folstein et al., 1975); NPI: Neuropsychiatric Inventory (Cummings et al., 1994); BEHAVE-AD: Behavioral Pathology in Alzheimer’s Disease Rating Scale (Reisberg et al., 2014).

### 4.2 Vascular cognitive impairment

Vascular cognitive impairment (VCI), attributable to cerebrovascular disease, encompasses a continuum ranging from mild cognitive impairment to dementia (Dichgans and Leys, 2017). Approximately 70% of patients with stroke exhibit cognitive impairment (Rost et al., 2022). Music therapy has been associated with improvements in cognitive function in VCI, potentially by activating cognitive networks, modulating the dopaminergic system, and promoting neural plasticity (Wang et al., 2025). Table 5 summarizes the efficacy of music therapy in VCI and the putative underlying mechanisms. A randomized controlled trial demonstrated that, after an acute unilateral stroke, early listening to singing was superior to instrumental music and audiobooks in improving cognitive and language functions. The instrumental music group induced right-hemisphere neuroplasticity in the aphasia subgroup, yet no corresponding cognitive benefits were observed, suggesting that the causal relationship between neural remodeling and functional recovery still needs to be explored (Sihvonen et al., 2020).

**TABLE 5:** Advances in the application of music therapy in improving cognitive impairment in VCI patients.

Disease	Intervention description	Intervention duration	Efficacy	Mechanism of action	References
Acute unilateral middle cerebral artery (MCA) stroke	Listening to self-selected music (predominantly popular music, with some jazz, folk, or classical) or audiobooks	6 months	Facilitated cognitive recovery (e.g., verbal memory and concentration) and helped prevent negative emotions (e.g., depression and confusion).	Activated bilateral temporal, frontal, and parietal cognitive networks and modulated dopaminergic emotion-related systems, potentially supporting neural remodeling.	(Särkämö et al., 2008)
				Voxel based morphometry (VBM) showed increased gray volume in the superior frontal gyrus, subgenual anterior cingulate cortex, and ventral striatum.	(Särkämö et al., 2014)
Ischemic stroke	Personalized music listening	3 months	Significantly improved MoCA scores (*p* = 0.027, mean difference = 3.21 points), particularly in the domains of delayed recall and orientation.	Activated broad cognitive brain regions, including the cerebellum, sensory cortex, and DMN; Evoked autobiographical memories via preferred music, involving the posteromedial cortex and posterior cingulate gyrus.	(Fan et al., 2024)
Chronic ischemic stroke	VRRS attention cognitive training combined with EMS	8 weeks	Significantly improved MoCA scores (*p* = 0.004, *r* = 0.64), intrinsic motivation, (*p* < 0.001, *r* = 0.84), depression (*p* < 0.001, *r* = 0.83), and anxiety (*p* = 0.003, *r* = 0.66).	Stabilized arousal levels and enhanced task focus; modulated limbic-prefrontal pathways; reduced heart rate and promoted autonomic balance.	(De Luca et al., 2025)
Acute unilateral stroke	Listening to self-selected vocal music, instrumental music, or audiobooks	2 months	Enhanced verbal memory (*p* = 0.002, η^2^ = 0.172) and language recovery, particularly in patients with aphasia (*p* < 0.001, η^2^ = 0.588); vocal music showed the greatest therapeutic effect.	Increased left temporal gray matter volume and DMN functional connectivity.	(Sihvonen et al., 2020)

MoCA, Montreal Cognitive Assessment (Nasreddine et al., 2005); VRRS, Virtual Reality Rehabilitation System; EMS, Emotional Music Stimulation.

### 4.3 Parkinson’s disease cognitive impairment (PD-MCI)

Parkinson’s disease (PD) is a progressive neurodegenerative disorder characterized by tremor and bradykinesia; most patients also present with cognitive deficits (Hayes, 2019; Bloem et al., 2021). Gait disturbances often coexist with cognitive decline, and freezing of gait is significantly associated with poorer global cognition, executive function, attention, working memory, and visuospatial ability (Monaghan et al., 2023). Executive dysfunction is an independent risk factor for falls (Pelicioni et al., 2021). NMT has demonstrated dual benefits for gait and cognition in patients with PD (Bella et al., 2015), with specific applications detailed in Table 6. In a preliminary randomized controlled trial (*n* = 12) involving patients with Parkinson’s disease, Park and Kim (2021) examined the effects of dual-task–based rhythmic-cueing drum playing. The intervention was associated with a large effect size on the Stroop Color–Word Test (*d* = 1.04). Accordingly, the authors suggested that bimanual drum playing may improve inhibitory control in this population. However, the between-group interaction did not reach statistical significance (*p* = 0.100); therefore, this conclusion requires validation in studies with larger sample sizes.

**TABLE 6:** Advances in the application of music therapy in improving cognitive impairment in PD-MCI patients.

Disease	Intervention description	Intervention duration		Efficacy	Mechanism of action	References
Interactive RAS and fixed RAS	Rhythmic Speech and Language Therapy (rSLT) and Rhythmic Balance and Movement Training (rBMT)	4 weeks	Cognitive function	rSLT improved working memory (*p* < 0.01, Cohen’s *d* = 0.94) and word retrieval (*p* < 0.01, Cohen’s *d* = 1.04). rBMT showed no significant cognitive gains but slowed cognitive decline.	Rhythm, as a salient stimulus, broadly activates brain networks (auditory, motor, memory [hippocampus], emotional [limbic], and language systems), thereby enhancing therapeutic engagement.	(Roesch et al., 2021)
	AMI: high-intensity group piano training	10 days		Significantly improved cognitive control, as evidenced by reduced Stroop task error rates (*p* = 0.03, *r* = 0.34) and increased musical self-efficacy (*p* = 0.001, *r* = 0.48).	Activated striato-thalamo-cortical networks, thereby bolstering fine motor skills and executive planning.	(Bugos et al., 2021)
	AMI: music production, singing, and dancing	24 weeks		Executive function and memory improved in the short term, but the effects dissipated after the intervention ended.	Stimulated and trained core executive functions, including attention, cognitive flexibility, processing speed, and working memory.	(Spina et al., 2016)
	Dual-task-based drumming performance with rhythmic cueing (DPRC)	12 weeks		The large effect (Cohen’s *d* = 1.04) for inhibitory control was non-significant (*p* = 0.100).	Rhythmic cues enhanced auditory–motor interactions, thereby reducing dual-task interference.	(Park and Kim, 2021)
	An autonomous closed-loop RAS automatically adjusted music tempo based on real-time gait parameters.	6 weeks		Significantly enhance walking intensity (*p* = 0.001, Cohen’s *d* = 1.2), increase daily step count (*p* = 0.003, Cohen’s *d* = 1.08), and reduce gait variability (*p* = 0.029, Cohen’s *d* = 0.79).	Auditory–motor network synchronization bypassed dysfunctional basal ganglia circuits, thereby improving motor rhythmicity and stability.	(Porciuncula et al., 2025)
	Interactive RAS and fixed RAS	6 months		Restored healthy 1/f fractal scaling, thereby enhancing gait and perceptual stability.	Stabilized the internal rhythm-generation system via mutual entrainment, thereby restoring basal ganglia function.	(Hove et al., 2012)
	BeatWalk is a smartphone application that synchronizes music tempo with step frequency in real time and progressively increases tempo to 10% above the user’s spontaneous cadence.	4 weeks		Significantly improved cadence, velocity, distance (*p* ≤ 0.01), and stride length (*p* = 0.04) on the 6-min walk test.	Used interactive beat-based entrainment to induce spontaneous synchronization, thereby mitigating dual-task interference and enhancing gait motivation and rhythmicity.	(Cochen De Cock et al., 2021)

### 4.4 Mild cognitive impairment

Mild cognitive impairment (MCI) lies between normal aging and dementia and is characterized by persistent memory decline that does not substantially affect activities of daily living; it also carries a risk of progression to dementia (Mian et al., 2024). Music therapy has shown considerable potential to ameliorate overall cognitive functioning in individuals with MCI (Lai et al., 2020). Specific applications are detailed in Table 7. In MCI, normal and damaged brain tissue coexist; however, no existing study has clarified whether the cognitive improvement derived from music interventions originates from compensatory recruitment of normal brain areas or from repair of damaged regions. Future studies require imaging markers to dissociate and observe the responses of the two tissue types.

**TABLE 7:** Advances in the application of music therapy in improving cognitive impairment in MCI patients.

Disease	Intervention description	Intervention duration	Efficacy	Mechanism of action	References
MCI	Multitask movement–music therapy	12 weeks	Significantly improved executive function, particularly motor programming (*p* = 0.021, *r* = 0.414) and overall frontal lobe cognitive performance (*p* < 0.05).	Activated the prefrontal cortex, increasing cerebral blood flow and functional connectivity.	(Shimizu et al., 2018)
	Music-and-movement intervention: rhythmic activities, music listening and song recognition, instrumental performance, and singing	12 weeks	Significant improvements in global cognition, attention, immediate and delayed memory, and executive function (*P* ≤ 0.007).	Activated the prefrontal cortex, increased cerebral blood flow, and promoted the formation of new neural networks.	(Domínguez-Chávez et al., 2019)
	RMI: listening to personalized music, group discussions, and progressive muscle relaxation training	8 weeks	Significantly improved the total MoCA score (*P* < 0.001, mean difference = 2.63, 95% CI: 2.09–3.17), with a medium effect size (Cohen’s *d* = 0.40).	Promoted psychophysiological relaxation through rhythmic and soothing properties, stimulated autobiographical memories, and may have improved neural function by upregulating BDNF levels.	(Xue et al., 2023)

### 4.5 Traumatic brain injury

Traumatic brain injury (TBI), resulting from external mechanical forces, frequently leads to cognitive impairment, with executive dysfunction being particularly prominent (Menon et al., 2010; Jeffay et al., 2023). Music therapy has demonstrated positive effects in ameliorating executive function in patients with TBI (Mishra et al., 2021; Alashram et al., 2025). Table 8 details the therapeutic efficacy and the putative underlying mechanisms through which music therapy improves executive function after TBI. Preclinical evidence supports these clinical observations. In a rat model of TBI induced by controlled cortical impact (CCI), daily exposure to Baroque classical music for 1 month significantly improved spatial learning and memory (*p* = 0.0007). This effect was associated with upregulation of hippocampal brain-derived neurotrophic factor, modulation of neuroinflammatory responses, and a reduction in cortical lesion volume (Moschonas et al., 2023). Interestingly, after receiving music therapy, TBI patients showed decreased connectivity between the DMN and the sensorimotor network, and the magnitude of this decrease was associated with improvements in executive function (Martínez-Molina et al., 2021). In populations with mild cognitive impairment and dementia, the meta-analysis by Mulia et al. (2026) hypothesized that cognitive benefits might be associated with enhanced interactions between the DMN and the task-positive network. These opposing modulation directions suggest that the effect of music therapy on the DMN may be regulated by baseline network states.

**TABLE 8:** Advances in the application of music therapy in improving cognitive impairment in TBI patients.

Disease	Intervention description	Intervention duration	Efficacy	Mechanism of action	References
TBI (mild)	NMT: a structured and standardized piano training curriculum	8 weeks	Significant improvement in cognitive function (*p* < 0.001); enhancement of subjective wellbeing and social behavior.	Enhanced functional connectivity within OFC networks (e.g., left anterior and posterior OFC, right OFC, etc.) was observed; repetitive practice strengthened synaptic connections; Goal attainment and perceived mastery increased dopamine release.	(Vik et al., 2018; Vik et al., 2019)
TBI (Moderate to severe)	NMT: rhythmic training, structured cognitive–motor training, and assisted music performance	3 months	Executive function improved significantly, as indicated by increased FAB scores (*p* = 0.021, *r* = 0.46).	Promoted white-matter structural remodeling, reflected by increased quantitative anisotropy in key regions (e.g., the right dorsal pathway, corpus callosum, thalamic radiation, and corticostriatal tract).	(Sihvonen et al., 2022)
			Executive function (*p* = 0.045, η^2^ = 0.093) and task-switching ability (*p* = 0.032, η^2^ = 0.112) improved significantly; only executive function was maintained at follow-up (*p* = 0.003, η^2^ = 0.328).	Promoted plasticity in prefrontal gray-matter structure, evidenced by increased gray-matter volume in the right inferior frontal gyrus.	(Siponkoski et al., 2020)
			Improved general executive function (*p* = 0.003, *r* = -0.559) and self-monitoring (*p* = 0.013, *r* = 0.485), as assessed by neuropsychological tests.	Enhanced resting-state network connectivity (FPN–DAN, FPN–SM, DAN–VIS), while reducing within-FPN hyperconnectivity and DMN-SM hyperconnectivity to optimize network efficiency.	(Martínez-Molina et al., 2022)

FAB, Frontal Assessment Battery (Dubois et al., 2000); OFC, Orbitofrontal Cortex; FPN, Frontoparietal Network; DAN, Dorsal Attention Network; SM, Sensorimotor Metwork; VIS, Visual Network.

## 5 Summary and outlook

Grounded in neuroplasticity theory, this article systematically reviews the mechanisms of action, technical frameworks, and clinical evidence supporting music therapy in the rehabilitation of cognitive impairment. Research indicates that music therapy may improve both cognitive function and neuropsychiatric symptoms across a range of cognitive disorders—including AD, VCI, PD-MCI, MCI, and TBI—via multilevel mechanisms, such as upregulating BDNF, promoting hippocampal neurogenesis, and optimizing brain-network connectivity. However, clinical efficacy varies by disease stage. Music therapy appears to yield the greatest cognitive improvements in patients with MCI and early-stage AD. By contrast, in moderate to severe AD, the primary clinical role of music therapy shifts toward alleviating neuropsychiatric symptoms, underscoring the importance of early intervention. Additionally, AMI and RMI appear to exert distinct therapeutic effects. AMI emphasizes cognitive–motor training and improvements in executive function, which may be related to multisensory integration induced by active participation. RMI primarily targets emotional and stress-related responses, potentially via activation of reward circuitry and modulation of the autonomic nervous system. However, direct evidence supporting synergistic effects of combining these two modalities remains lacking.

The field continues to face substantial challenges: (1) An explanatory gap persists at the mechanistic level: evidence for alterations in molecular pathways is derived largely from animal studies, whereas evidence for brain-network plasticity is based primarily on neuroimaging; however, validated causal chains linking these levels of evidence remain lacking, making it difficult to directly attribute clinical efficacy to specific neuroplastic changes. (2) Influence of Multicultural and Musical Backgrounds: Music therapy is inherently highly individualized, and its efficacy is strongly shaped by patients’ musical backgrounds and emotional associations. However, the cited clinical studies largely involve Chinese populations and healthcare settings and typically use standardized intervention protocols to evaluate average treatment effects at the group level. Consequently, these positive findings are difficult to translate into replicable intervention guidelines. (3) Outcome Measurement Heterogeneity: The included studies used different assessment instruments, including the MMSE, MoCA, NPI, FAB, and BEHAVE-AD. Because these instruments differ in measurement properties and in sensitivity across disease stages, this heterogeneity in outcomes reduced the comparability of findings across studies and posed a substantial barrier to systematic cross-study integration. (4) Intervention models remain relatively homogeneous at the clinical-translation stage: strategies are typically implemented as fixed protocols and lack dynamic adjustment mechanisms informed by real-time patient feedback.

Future research should focus on revealing the synergistic integration pathways among multilevel mechanisms in music interventions, namely BDNF pathway activation, optimization of the salience and control networks, and preservation of white matter integrity, in order to elucidate the potential mechanisms through which these processes drive cognitive plasticity. Furthermore, incorporating real-time monitoring and feedback mechanisms into clinical trials, together with establishing a tiered, phased, and adaptive intervention framework, may render music therapy a “quantifiable science”. Meanwhile, by preserving its unique role as a carrier of human emotion, it is expected to be truly integrated into a precision rehabilitation system for cognitive disorders.

## References

[B1] Abrahams T. P. van Dooren J. C. (2018). Musical attention control training (MACT) in secure residential youth care: A randomised controlled pilot study. *Arts Psychother.* 57 80–87. 10.1016/j.aip.2017.10.008

[B2] Alashram A. R. Janada Q. Ghrear T. Annino G. (2025). Role of music therapy in improving cognitive function post-traumatic brain injury: A systematic review. *Appl. Neuropsychol. Adult* 32 1486–1495. 10.1080/23279095.2023.2228951 37389826

[B3] Andrews E. Eierud C. Banks D. Harshbarger T. Michael A. Rammell C. (2023). DTI analysis of white matter integrity and cognitive brain reserve in lifelong musicians and controls. *J. Psychiatry Psychiatr. Disord.* 7 80–88. 10.26502/jppd.2572-519X0187

[B4] Bai W. Chen P. Cai H. Zhang Q. Su Z. Cheung T. et al. (2022). Worldwide prevalence of mild cognitive impairment among community dwellers aged 50 years and older: A meta-analysis and systematic review of epidemiology studies. *Age Ageing* 51:afac173. 10.1093/ageing/afac173 35977150

[B5] Basile G. A. Bertino S. Nozais V. Bramanti A. Ciurleo R. Anastasi G. P. et al. (2022). White matter substrates of functional connectivity dynamics in the human brain. *Neuroimage* 258:119391. 10.1016/j.neuroimage.2022.119391 35716842

[B6] Bella S. D. Benoit C.-E. Farrugia N. Keller P. E. Obrig H. Mainka S. et al. (2017). Gait improvement via rhythmic stimulation in Parkinson’s disease is linked to rhythmic skills. *Sci. Rep.* 7:42005. 10.1038/srep42005 28233776 PMC5324039

[B7] Bella S. D. Benoit C. Farrugia N. Schwartze M. Kotz S. A. (2015). Effects of musically cued gait training in Parkinson’s disease: beyond a motor benefit. *Ann. N. Y. Acad. Sci.* 1337, 77–85. 10.1111/nyas.12651 25773620

[B8] Bianco R. Novembre G. Ringer H. Kohler N. Keller P. E. Villringer A. et al. (2021). Lateral prefrontal cortex is a hub for music production from structural rules to movements. *Cereb. Cortex* 32 3878–3895. 10.1093/cercor/bhab454 34965579 PMC9476625

[B9] Bleibel M. El Cheikh A. Sadier N. S. Abou-Abbas L. (2023). The effect of music therapy on cognitive functions in patients with Alzheimer’s disease: A systematic review of randomized controlled trials. *Alzheimers Res. Ther.* 15:65. 10.1186/s13195-023-01214-9 36973733 PMC10041788

[B10] Bloem B. R. Okun M. S. Klein C. (2021). Parkinson’s disease. *Lancet* 397 2284–2303. 10.1016/S0140-6736(21)00218-X 33848468

[B11] Bombonato C. Del Lucchese B. Ruffini C. Di Lieto M. C. Brovedani P. Sgandurra G. et al. (2024). Far transfer effects of trainings on executive functions in neurodevelopmental disorders: A systematic review and metanalysis. *Neuropsychol. Rev.* 34 98–133. 10.1007/s11065-022-09574-z 36633797 PMC10920464

[B12] Bugos J. A. Lesiuk T. Nathani S. (2021). Piano training enhances stroop performance and musical self-efficacy in older adults with Parkinson’s disease. *Psychol. Music* 49 615–630. 10.1177/0305735619888571

[B13] Calsolaro V. Femminella G. D. Rogani S. Esposito S. Franchi R. Okoye C. et al. (2021). Behavioral and psychological symptoms in dementia (BPSD) and the use of antipsychotics. *Pharmaceuticals* 14:246. 10.3390/ph14030246 33803277 PMC8002184

[B14] Cannon J. J. Patel A. D. (2021). How beat perception coopts motor neurophysiology. *Trends Cogn. Sci.* 25 137–150. 10.1016/j.tics.2020.11.002 33353800 PMC9440376

[B15] Castro M. L’héritier F. Plailly J. Saive A.-L. Corneyllie A. Tillmann B. et al. (2020). Personal familiarity of music and its cerebral effect on subsequent speech processing. *Sci. Rep.* 10:14854. 10.1038/s41598-020-71855-5 32908227 PMC7481778

[B16] Chafee M. V. Heilbronner S. R. (2022). Prefrontal cortex. *Curr. Biol.* 32 R346–R351. 10.1016/j.cub.2022.02.071 35472417 PMC9832551

[B17] Chatterjee D. Hegde S. Thaut M. (2021). Neural plasticity: The substratum of music-based interventions in neurorehabilitation. *Neurorehabilitation* 48 155–166. 10.3233/NRE-208011 33579881 PMC7613141

[B18] Cheng H.-Y. Xie H.-X. Tang Q.-L. Yi L.-T. Zhu J.-X. (2024). Light and classical music therapies attenuate chronic unpredictable mild stress-induced depression via BDNF signaling pathway in mice. *Heliyon* 10:e34196. 10.1016/j.heliyon.2024.e34196 39071672 PMC11283034

[B19] Cochen De Cock V. Dotov D. Damm L. Lacombe S. Ihalainen P. Picot M. C. et al. (2021). BeatWalk: Personalized music-based gait rehabilitation in Parkinson’s disease. *Front. Psychol.* 12:655121. 10.3389/fpsyg.2021.655121 33981279 PMC8109247

[B20] Compère L. Charron S. Gallarda T. Rari E. Lion S. Nys M. et al. (2021). Gender identity better than sex explains individual differences in episodic and semantic components of autobiographical memory: An fMRI study. *Neuroimage* 225:117507. 10.1016/j.neuroimage.2020.117507 33127480

[B21] Cummings J. L. Mega M. Gray K. Rosenberg-Thompson S. Carusi D. A. Gornbein J. (1994). The neuropsychiatric inventory: Comprehensive assessment of psychopathology in dementia. *Neurology* 44 2308–2314. 10.1212/WNL.44.12.2308 7991117

[B22] de Aquino M. P. B. Verdejo-Román J. Pérez-García M. Pérez-García P. (2019). Different role of the supplementary motor area and the insula between musicians and non-musicians in a controlled musical creativity task. *Sci. Rep.* 9:13006. 10.1038/s41598-019-49405-5 31506553 PMC6736976

[B23] De L’Etoile S. Jones C. (2025). “’Associative mood and memory training,” in *Handbook of Neurologic Music Therapy*, eds Thaut M. H. Hömberg V. (Oxford: Oxford University Press), 397–412.

[B24] De Luca R. Impellizzeri F. Corallo F. Calderone A. Calapai R. Mirabile A. et al. (2025). Targeting cognition and behavior post-stroke: Combined emotional music stimulation and virtual attention training in a quasi-randomized study. *Brain Sci.* 15:1168. 10.3390/brainsci15111168 41300175 PMC12650013

[B25] de Souza Almeida R. Faria-Jr A. Klein R. M. (2021). On the origins and evolution of the Attention Network Tests. *Neurosci. Biobehav. Rev.* 126, 560–572. 10.1016/j.neubiorev.2021.02.028 33766674

[B26] Dichgans M. Leys D. (2017). Vascular cognitive impairment. *Circ. Res.* 120 573–591. 10.1161/circresaha.116.308426 28154105

[B27] Domínguez-Chávez C. J. Murrock C. J. Guerrero P. I. C. Salazar-González B. C. (2019). Music therapy intervention in community-dwelling older adults with mild cognitive impairment: A pilot study. *Geriatr. Nurs.* 40 614–619. 10.1016/j.gerinurse.2019.06.004 31277962

[B28] Dubois B. Slachevsky A. Litvan I. Pillon B. (2000). The FAB: A frontal assessment battery at bedside. *Neurology* 55 1621–1626. 10.1212/wnl.55.11.1621 11113214

[B29] Emmery L. Hackney M. E. Kesar T. McKay J. L. Rosenberg M. C. (2023). An integrated review of music cognition and rhythmic stimuli in sensorimotor neurocognition and neurorehabilitation. *Ann. N. Y. Acad. Sci.* 1530 74–86. 10.1111/nyas.15079 37917153 PMC10841443

[B30] Fan L. Quijano-Ruiz A. Wang C. Zhao H. Wang D. Wu H. et al. (2024). Effects of personalized music listening on post-stroke cognitive impairment: A randomized controlled trial. *Complement. Ther. Clin. Pract.* 57:101885. 10.1016/j.ctcp.2024.101885 39098085

[B31] Fischer C. E. Churchill N. Leggieri M. Vuong V. Tau M. Fornazzari L. R. et al. (2021). Long-known music exposure effects on brain imaging and cognition in early-stage cognitive decline: A pilot study. *J. Alzheimers Dis.* 84 819–833. 10.3233/JAD-210610 34602475

[B32] Folstein M. F. Folstein S. E. McHugh P. R. (1975). “Mini-mental state”: A practical method for grading the cognitive state of patients for the clinician. *J. Psychiatr. Res.* 12 189–198. 10.1016/0022-3956(75)90026-6 1202204

[B33] Friend D. Kravitz A. (2014). Working together: Basal ganglia pathways in action selection. *Trends Neurosci.* 37 301–303. 10.1016/j.tins.2014.04.004 24816402 PMC4041812

[B34] Frühholz S. Trost W. Grandjean D. (2014). The role of the medial temporal limbic system in processing emotions in voice and music. *Prog. Neurobiol.* 123 1–17. 10.1016/j.pneurobio.2014.09.003 25291405

[B35] Gómez Gallego M. Gómez García J. (2017). Musicoterapia en la enfermedad de Alzheimer: Efectos cognitivos, psicológicos y conductuales. *Neurología* 32 300–308. 10.1016/j.nrl.2015.12.003 26896913

[B36] Gómez-Gallego M. Gómez-Gallego J. C. Gallego-Mellado M. García-García J. (2021). Comparative efficacy of active group music intervention versus group music listening in Alzheimer’s disease. *Int. J. Environ. Res. Public Health* 18:8067. 10.3390/ijerph18158067 34360360 PMC8345612

[B37] Guo Y. Zheng H. Long J. (2023). Gating at cortical level contributes to auditory-motor synchronization during repetitive finger tapping. *Cereb. Cortex* 33 6198–6206. 10.1093/cercor/bhac495 36563001

[B38] Hakvoort L. Jones C. Gardiner J. C. Thaut M. H. (2025). “Musical attention control training (MACT)^§^,” in *Handbook of Neurologic Music Therapy*, eds Thaut M. H. Hömberg V. (Oxford: Oxford University Press), 322–337.

[B39] Harding E. E. Kim J. C. Demos A. P. Roman I. R. Tichko P. Palmer C. et al. (2025). Musical neurodynamics. *Nat. Rev. Neurosci.* 26 293–307. 10.1038/s41583-025-00915-4 40102614

[B40] Hayes M. T. (2019). Parkinson’s disease and Parkinsonism. *Am. J. Med.* 132 802–807. 10.1016/j.amjmed.2019.03.001 30890425

[B41] Hegde S. Thaut M. H. Gardiner J. C. (2025). “Musical executive function training (MEFT)^§^,” in *Handbook of Neurologic Music Therapy*, eds Thaut M. H. Hömberg V. (Oxford: Oxford University Press), 352–370.

[B42] Herrojo Ruiz M. Maess B. Altenmüller E. Curio G. Nikulin V. V. (2017). Cingulate and cerebellar beta oscillations are engaged in the acquisition of auditory-motor sequences. *Hum. Brain Mapp.* 38 5161–5179. 10.1002/hbm.23722 28703919 PMC6866917

[B43] Hove M. J. Suzuki K. Uchitomi H. Orimo S. Miyake Y. (2012). Interactive rhythmic auditory stimulation reinstates natural 1/f timing in gait of Parkinson’s patients. *PLoS One* 7:e32600. 10.1371/journal.pone.0032600 22396783 PMC3292577

[B44] Jacobsen J.-H. Stelzer J. Fritz T. H. Chételat G. La Joie R. Turner R. (2015). Why musical memory can be preserved in advanced Alzheimer’s disease. *Brain* 138 2438–2450. 10.1093/brain/awv135 26041611

[B45] Jeffay E. Ponsford J. Harnett A. Janzen S. Patsakos E. Douglas J. et al. (2023). INCOG 2.0 guidelines for cognitive rehabilitation following traumatic brain injury, part III: Executive functions. *J. Head Trauma Rehabil.* 38 52–64. 10.1097/HTR.0000000000000834 36594859

[B46] Jia L. Du Y. Chu L. Zhang Z. Li F. Lyu D. et al. (2020). Prevalence, risk factors, and management of dementia and mild cognitive impairment in adults aged 60 years or older in China: A cross-sectional study. *Lancet Public Health* 5 e661–e671. 10.1016/S2468-2667(20)30185-7 33271079

[B47] Jones C. Richard N. Thaut M. (2021). Investigating music-based cognitive rehabilitation for individuals with moderate to severe chronic acquired brain injury: A feasibility experiment. *Neurorehabilitation* 48 209–220. 10.3233/NRE-208015 33664158

[B48] Juslin P. N. Västfjäll D. (2008). Emotional responses to music: The need to consider underlying mechanisms. *Behav. Brain Sci.* 31 559–575; discussion 575–621. 10.1017/S0140525X08005293 18826699

[B49] Kensinger E. A. Ford J. H. (2020). Retrieval of emotional events from memory. *Annu. Rev. Psychol.* 71 251–272. 10.1146/annurev-psych-010419-051123 31283426

[B50] Kerns J. G. Cohen J. D. MacDonald A. W. Cho R. Y. Stenger V. A. Carter C. S. (2004). Anterior cingulate conflict monitoring and adjustments in control. *Science* 303 1023–1026. 10.1126/science.1089910 14963333

[B51] King J. B. Jones K. G. Goldberg E. Rollins M. MacNamee K. Moffit C. et al. (2019). Increased functional connectivity after listening to favored music in adults with Alzheimer dementia. *J. Prev. Alzheimers Dis.* 6 56–62. 10.14283/jpad.2018.19 30569087 PMC12280822

[B52] Koshimori Y. Thaut M. H. (2023). Rhythmic auditory stimulation as a potential neuromodulator for Parkinson’s disease. *Parkinsonism Relat. Disord.* 113:105459. 10.1016/j.parkreldis.2023.105459 37277293

[B53] Kunikullaya U. K. Pranjiæ M. Rigby A. Pallás-Ferrer I. Anand H. Kunnavil R. et al. (2025). The molecular basis of music-induced neuroplasticity in humans: A systematic review. *Neurosci. Biobehav. Rev.* 175:106219. 10.1016/j.neubiorev.2025.106219 40412457

[B54] Lai X. Wen H. Li Y. Lu L. Tang C. (2020). The comparative efficacy of multiple interventions for mild cognitive impairment in Alzheimer’s disease: A Bayesian network meta-analysis. *Front. Aging Neurosci.* 12:121. 10.3389/fnagi.2020.00121 32581760 PMC7289916

[B55] Lakatos P. Karmos G. Mehta A. D. Ulbert I. Schroeder C. E. (2008). Entrainment of neuronal oscillations as a mechanism of attentional selection. *Science* 320 110–113. 10.1126/science.1154735 18388295

[B56] Lata F. Kourtesis I. (2021). Listening to music as a stress management tool. *Eur. Psychiatry* 64:S609. 10.1192/j.eurpsy.2021.1621

[B57] Li C.-H. Liu C.-K. Yang Y.-H. Chou M.-C. Chen C.-H. Lai C.-L. (2015). Adjunct effect of music therapy on cognition in Alzheimer’s disease in Taiwan: A pilot study. *Neuropsychiatr. Dis. Treat.* 11 291–296. 10.2147/NDT.S73928 25678794 PMC4322884

[B58] Liu M.-N. Liou Y.-J. Wang W.-C. Su K.-C. Yeh H.-L. Lau C. et al. (2020). Group music intervention using percussion instruments to reduce anxiety among elderly male veterans with Alzheimer disease. *Med. Sci. Monit.* 27:e928714. 10.12659/MSM.928714 33611334 PMC7905960

[B59] Liu Y. Zhao C. Sander-Thömmes T. Yang T. Bao Y. (2018). Beta oscillation is an indicator for two patterns of sensorimotor synchronization. *Psych J.* 13 347–354. 10.1002/pchj.696 37905907 PMC11169746

[B60] Lyu J. Zhang J. Mu H. Li W. Champ M. Xiong Q. et al. (2018). The effects of music therapy on cognition, psychiatric symptoms, and activities of daily living in patients with Alzheimer’s disease. *J. Alzheimers Dis.* 64 1347–1358. 10.3233/JAD-180183 29991131

[B61] Martí,nez-Molina N. Siponkoski S. Särkämö T. (2022). Cognitive efficacy and neural mechanisms of music-based neurological rehabilitation for traumatic brain injury. *Ann. N. Y. Acad. Sci.* 1515 20–32. 10.1111/nyas.14800 35676218 PMC9796942

[B62] Martínez-Molina N. Siponkoski S.-T. Kuusela L. Laitinen S. Holma M. Ahlfors M. et al. (2021). Resting-state network plasticity induced by music therapy after traumatic brain injury. *Neural Plast.* 2021:6682471. 10.1155/2021/6682471 33763126 PMC7964116

[B63] McGrattan A. M. Pakpahan E. Siervo M. Mohan D. Reidpath D. D. Prina M. et al. (2022). Risk of conversion from mild cognitive impairment to dementia in low- and middle-income countries: A systematic review and meta-analysis. *Alzheimers Dement.* 8:e12267. 10.1002/trc2.12267 35310524 PMC8918697

[B64] Meilán García J. J. Iodice R. Carro J. Sánchez J. A. Palmero F. Mateos A. M. (2012). Improvement of autobiographic memory recovery by means of sad music in Alzheimer’s disease type dementia. *Aging Clin. Exp. Res.* 24 227–232. 10.3275/7874 21778809

[B65] Menon D. K. Schwab K. Wright D. W. Maas A. I. (2010). Position statement: Definition of traumatic brain injury. *Arch. Phys. Med. Rehabil.* 91 1637–1640. 10.1016/j.apmr.2010.05.017 21044706

[B66] Merenstein J. L. Song A. W. Madden D. J. (2025). High-resolution structural connectivity mediates age-related differences in functional connectivity and fluid cognition. *Brain Commun.* 7:fcaf376. 10.1093/braincomms/fcaf376 41113674 PMC12528985

[B67] Mian M. Tahiri J. Eldin R. Altabaa M. Sehar U. Reddy P. H. (2024). Overlooked cases of mild cognitive impairment: Implications to early Alzheimer’s disease. *Ageing Res. Rev.* 98:102335. 10.1016/j.arr.2024.102335 38744405 PMC11180381

[B68] Mishra R. Florez-Perdomo W. A. Shrivatava A. Chouksey P. Raj S. Moscote-Salazar L. R. et al. (2021). Role of music therapy in traumatic brain injury: A systematic review and meta-analysis. *World Neurosurg.* 146 197–204. 10.1016/j.wneu.2020.10.130 33130286

[B69] Mojtabavi H. Saghazadeh A. Valenti V. E. Rezaei N. (2020). Can music influence cardiac autonomic system? A systematic review and narrative synthesis to evaluate its impact on heart rate variability. *Complement. Ther. Clin. Pract.* 39:101162. 10.1016/j.ctcp.2020.101162 32379689

[B70] Monaghan A. S. Gordon E. Graham L. Hughes E. Peterson D. S. Morris R. (2023). Cognition and freezing of gait in Parkinson’s disease: A systematic review and meta-analysis. *Neurosci. Biobehav. Rev.* 147:105068. 10.1016/j.neubiorev.2023.105068 36738813

[B71] Moreta M. P.-G. Burgos-Alonso N. Torrecilla M. Marco-Contelles J. Bruzos-Cidón C. (2021). Efficacy of acetylcholinesterase inhibitors on cognitive function in Alzheimer’s disease. Review of reviews. *Biomedicines* 9:1689. 10.3390/biomedicines9111689 34829917 PMC8615650

[B72] Morozova A. Zorkina Y. Abramova O. Pavlova O. Pavlov K. Soloveva K. et al. (2022). Neurobiological highlights of cognitive impairment in psychiatric disorders. *Int. J. Mol. Sci.* 23:1217. 10.3390/ijms23031217 35163141 PMC8835608

[B73] Moschonas E. H. Ranellone T. S. Vozzella V. J. Rennerfeldt P. L. Bondi C. O. Annas E. M. et al. (2023). Efficacy of a music-based intervention in a preclinical model of traumatic brain injury: An initial foray into a novel and non-pharmacological rehabilitative therapy. *Exp. Neurol.* 369:114544. 10.1016/j.expneurol.2023.114544 37726048 PMC10591861

[B74] Mulia G. J. Chiang Y. H. Maringka S. G. Wu J. C. Ma H. P. Ou J. C. et al. (2026). Cognitive enhancement through music therapy: Meta-analytic evidence across clinical population. *Front. Public Health* 13:1735470. 10.3389/fpubh.2025.1735470 41584142 PMC12823501

[B75] Nasreddine Z. S. Phillips N. A. Bédirian V. Charbonneau S. Whitehead V. Collin I. et al. (2005). The Montreal Cognitive Assessment, MoCA: A brief screening tool for mild cognitive impairment. *J. Am. Geriatr. Soc.* 53 695–699. 10.1111/j.1532-5415.2005.53221.x 15817019

[B76] Ooishi Y. Mukai H. Watanabe K. Kawato S. Kashino M. (2017). Increase in salivary oxytocin and decrease in salivary cortisol after listening to relaxing slow-tempo and exciting fast-tempo music. *PLoS One* 12:e0189075. 10.1371/journal.pone.0189075 29211795 PMC5718605

[B77] Paquette S. Takerkart S. Saget S. Peretz I. Belin P. (2018). Cross-classification of musical and vocal emotions in the auditory cortex. *Ann. N. Y. Acad. Sci.* 1423 329–337. 10.1111/nyas.13666 29741242

[B78] Park J.-K. Kim S. J. (2021). Dual-task-based drum playing with rhythmic cueing on motor and attention control in patients with Parkinson’s disease: A preliminary randomized study. *Int. J. Environ. Res. Public Health* 18:10095. 10.3390/ijerph181910095 34639396 PMC8508067

[B79] Pasiali V. LaGasse A. B. Penn S. L. (2014). The effect of musical attention control training (MACT) on attention skills of adolescents with neurodevelopmental delays: A pilot study. *J. Music Ther.* 51 333–354. 10.1093/jmt/thu030 25504177

[B80] Pelicioni P. H. S. Menant J. C. Henderson E. J. Latt M. D. Brodie M. A. Lord S. R. (2021). Mild and marked executive dysfunction and falls in people with Parkinson’s disease. *Braz. J. Phys. Ther.* 25 437–443. 10.1016/j.bjpt.2020.11.005 33349526 PMC8353304

[B81] Porciuncula F. Cavanaugh J. T. Zajac J. Wendel N. Baker T. Arumukhom Revi D. et al. (2025). Amplifying walking activity in Parkinson’s disease through autonomous music-based rhythmic auditory stimulation: Randomized controlled trial. *NPJ Park. Dis.* 11:100. 10.1038/s41531-025-00952-x 40301366 PMC12041193

[B82] Putkinen V. Nazari-Farsani S. Seppälä K. Karjalainen T. Sun L. Karlsson H. K. et al. (2021). Decoding music-evoked emotions in the auditory and motor cortex. *Cereb. Cortex* 31 2549–2560. 10.1093/cercor/bhaa373 33367590

[B83] Raglio A. (2023). A novel music-based therapeutic approach: The therapeutic music listening. *Front. Hum. Neurosci.* 17:1204593. 10.3389/fnhum.2023.1204593 37520927 PMC10375023

[B84] Ranganath C. (2022). What is episodic memory and how do we use it? *Trends Cogn. Sci.* 26 1059–1061. 10.1016/j.tics.2022.09.023 36335016

[B85] Reisberg B. Monteiro I. Torossian C. Auer S. Shulman M. B. Ghimire S. et al. (2014). The BEHAVE-AD assessment system: A perspective, a commentary on new findings, and a historical review. *Dement. Geriatr. Cogn. Disord.* 38 89–146. 10.1159/000357839 24714384 PMC4216810

[B86] Roesch A. D. Gschwandtner U. Handabaka I. Meyer A. Taub E. Fuhr P. (2021). Effects of rhythmic interventions on cognitive abilities in Parkinson’s disease. *Dement. Geriatr. Cogn. Disord.* 50 372–386. 10.1159/000519122 34808624

[B87] Rost N. S. Brodtmann A. Pase M. P. van Veluw S. J. Biffi A. Duering M. et al. (2022). Post-stroke cognitive impairment and dementia. *Circ. Res.* 130 1252–1271. 10.1161/CIRCRESAHA.122.319951 35420911

[B88] Sachdev P. S. Blacker D. Blazer D. G. Ganguli M. Jeste D. V. Paulsen J. S. et al. (2014). Classifying neurocognitive disorders: The DSM-5 approach. *Nat. Rev. Neurol.* 10 634–642. 10.1038/nrneurol.2014.181 25266297

[B89] Sakamoto M. Ando H. Tsutou A. (2013). Comparing the effects of different individualized music interventions for elderly individuals with severe dementia. *Int. Psychogeriatr.* 25 775–784. 10.1017/S1041610212002256 23298693 PMC3605862

[B90] Salimpoor V. N. Benovoy M. Larcher K. Dagher A. Zatorre R. J. (2011). Anatomically distinct dopamine release during anticipation and experience of peak emotion to music. *Nat. Neurosci.* 14 257–262. 10.1038/nn.2726 21217764

[B91] Särkämö T. Ripollés P. Vepsäläinen H. Autti T. Silvennoinen H. M. Salli E. et al. (2014). Structural changes induced by daily music listening in the recovering brain after middle cerebral artery stroke: A voxel-based morphometry study. *Front. Hum. Neurosci.* 17:245. 10.3389/fnhum.2014.00245 24860466 PMC4029020

[B92] Särkämö T. Tervaniemi M. Laitinen S. Forsblom A. Soinila S. Mikkonen M. et al. (2008). Music listening enhances cognitive recovery and mood after middle cerebral artery stroke. *Brain* 131 866–876. 10.1093/brain/awn013 18287122

[B93] Scheltens P. De Strooper B. Kivipelto M. Holstege H. Chételat G. Teunissen C. E. et al. (2021). Alzheimer’s disease. *Lancet* 397 1577–1590. 10.1016/S0140-6736(20)32205-4 33667416 PMC8354300

[B94] Schlaug G. (2015). Musicians and music making as a model for the study of brain plasticity. *Prog. Brain Res.* 217 37–55. 10.1016/bs.pbr.2014.11.020 25725909 PMC4430083

[B95] Schneider L. Gossé L. Montgomery M. Wehmeier M. Villringer A. Fritz T. H. (2022). Components of active music interventions in therapeutic settings-present and future applications. *Brain Sci.* 12:622. 10.3390/brainsci12050622 35625009 PMC9139247

[B96] Segado M. Zatorre R. J. Penhune V. B. (2021). Effector-independent brain network for auditory-motor integration: FMRI evidence from singing and cello playing. *Neuroimage* 237:118128. 10.1016/j.neuroimage.2021.118128 33989814

[B97] Shi E. R. Zhang Q. (2020). A domain-general perspective on the role of the basal ganglia in language and music: Benefits of music therapy for the treatment of aphasia. *Brain Lang.* 206:104811. 10.1016/j.bandl.2020.104811 32442810

[B98] Shimizu N. Umemura T. Matsunaga M. Hirai T. (2018). Effects of movement music therapy with a percussion instrument on physical and frontal lobe function in older adults with mild cognitive impairment: A randomized controlled trial. *Aging Ment. Health* 22 1614–1626. 10.1080/13607863.2017.1379048 28937272

[B99] Shin J.-H. Jeong E. (2023). Virtual reality-based music attention training for acquired brain injury: A protocol for randomized cross-over trial. *Front. Neurol.* 14:1192181. 10.3389/fneur.2023.1192181 37638184 PMC10450247

[B100] Sihvonen A. J. Leo V. Ripollés P. Lehtovaara T. Ylönen A. Rajanaro P. et al. (2020). Vocal music enhances memory and language recovery after stroke: Pooled results from two RCTs. *Ann. Clin. Transl. Neurol.* 7 2272–2287. 10.1002/acn3.51217 33022148 PMC7664275

[B101] Sihvonen A. J. Siponkoski S.-T. Martínez-Molina N. Laitinen S. Holma M. Ahlfors M. et al. (2022). Neurological music therapy rebuilds structural connectome after traumatic brain injury: Secondary analysis from a randomized controlled trial. *J. Clin. Med.* 11:2184. 10.3390/jcm11082184 35456277 PMC9032739

[B102] Siponkoski S.-T. Martínez-Molina N. Kuusela L. Laitinen S. Holma M. Ahlfors M. et al. (2020). Music therapy enhances executive functions and prefrontal structural neuroplasticity after traumatic brain injury: Evidence from a randomized controlled trial. *J. Neurotrauma* 37 618–634. 10.1089/neu.2019.6413 31642408

[B103] Slade B. Williams B. Engelbrecht R. Ciorciari J. (2025). Improving executive functioning and reducing the risk of Alzheimer’s disease with music therapy: A narrative review of potential neural mechanisms. *J. Alzheimers Dis.* 105 319–330. 10.1177/13872877251327762 40123371 PMC12231918

[B104] Spina E. Barone P. Mosca L. L. Forges Davanzati R. Lombardi A. Longo K. et al. (2016). Music therapy for motor and nonmotor symptoms of Parkinson’s disease: A prospective, randomized, controlled, single-blinded study. *J. Am. Geriatr. Soc.* 64 e36–e39. 10.1111/jgs.14295 27458987

[B105] Sreeraj V. S. Shivakumar V. Sowmya S. Bose A. Nawani H. Narayanaswamy J. C. et al. (2019). Online theta frequency transcranial alternating current stimulation for cognitive remediation in schizophrenia: A case report and review of literature. *J. ECT* 35 139–143. 10.1097/YCT.0000000000000523 30024457

[B106] Svansdottir H. B. Snaedal J. (2006). Music therapy in moderate and severe dementia of Alzheimer’s type: A case-control study. *Int. Psychogeriatr.* 18 613–621. 10.1017/S1041610206003206 16618375

[B107] Tallon-Baudry C. (2012). On the neural mechanisms subserving consciousness and attention. *Front. Psychol.* 2:397. 10.3389/fpsyg.2011.00397 22291674 PMC3253412

[B108] Tewarie P. Hunt B. A. E. O’Neill G. C. Byrne A. Aquino K. Bauer M. et al. (2019). Relationships between neuronal oscillatory amplitude and dynamic functional connectivity. *Cereb. Cortex* 29 2668–2681. 10.1093/cercor/bhy136 29897408

[B109] Thaut C. P. Rice R. (2025). “Rhythmic auditory stimulation (RAS)^®^,” in *Handbook of Neurologic Music Therapy*, 2nd Edn, eds Thaut M. H. Hömberg V. (Oxford: Oxford University Press), 99–114.

[B110] Tsoi K. K. F. Chan J. Y. C. Ng Y.-M. Lee M. M. Y. Kwok T. C. Y. Wong S. Y. S. (2018). Receptive music therapy is more effective than interactive music therapy to relieve behavioral and psychological symptoms of dementia: A systematic review and meta-analysis. *J. Am. Med. Dir. Assoc.* 19 568–576.e3. 10.1016/j.jamda.2017.12.009 29396186

[B111] van Alphen R. Stams G. J. J. M. Hakvoort L. (2019). Musical attention control training for psychotic psychiatric patients: An experimental pilot study in a forensic psychiatric hospital. *Front. Neurosci.* 13:570. 10.3389/fnins.2019.00570 31231183 PMC6566130

[B112] Vik B. M. D. Skeie G. O. Specht K. (2019). Neuroplastic effects in patients with traumatic brain injury after music-supported therapy. *Front. Hum. Neurosci.* 25:177. 10.3389/fnhum.2019.00177 31293405 PMC6604902

[B113] Vik B. M. D. Skeie G. O. Vikane E. Specht K. (2018). Effects of music production on cortical plasticity within cognitive rehabilitation of patients with mild traumatic brain injury. *Brain Inj*. 32 634–643. 10.1080/02699052.2018.1431842 29388854

[B114] Vuust P. Heggli O. A. Friston K. J. Kringelbach M. L. (2022). Music in the brain. *Nat. Rev. Neurosci.* 23 287–305. 10.1038/s41583-022-00578-5 35352057

[B115] Wang J. Wang J. Wang Y. Chai Y. Li H. Miao D. et al. (2023). Music with different tones affects the development of brain nerves in mice in early life through BDNF and its downstream pathways. *Int. J. Mol. Sci.* 24:8119. 10.3390/ijms24098119 37175826 PMC10179650

[B116] Wang S. G. Cevasco-Trotter A. M. Silverman M. J. Yuan S. H. (2023). A narrative review of music therapy for neuropsychiatric symptoms in Alzheimer’s disease and rationale for protocolized music teletherapy. *Front. Med.* 10:1248245. 10.3389/fmed.2023.1248245 38076267 PMC10703166

[B117] Wang Z. Xue Y. Sun G. Yun J. Li Y. Chen Q. et al. (2025). Effects of music-supported therapy for depression and cognitive disorders in people living with stroke and its impact on quality of life: A systematic evaluation and meta-analysis. *Cerebrovasc. Dis.* 54 959–984. 10.1159/000543361 39761658

[B118] Wei Y. Qiao Z. (2024). Neurologic music therapy’s impact on neurological disorders. *J. Neurosci. Res.* 102:e70000. 10.1002/jnr.70000 39625180

[B119] Wu Z. Kong L. Zhang Q. (2022). Research progress of music therapy on gait intervention in patients with Parkinson’s disease. *Int. J. Environ. Res. Public Health* 19:9568. 10.3390/ijerph19159568 35954925 PMC9368619

[B120] Xia H. He Q. Chen A. (2022). Understanding cognitive control in aging: A brain network perspective. *Front. Aging Neurosci.* 14:1038756. 10.3389/fnagi.2022.1038756 36389081 PMC9659905

[B121] Xue B. Meng X. Liu Q. Luo X. (2023). The effect of receptive music therapy on older adults with mild cognitive impairment and depression: A randomized controlled trial. *Sci. Rep.* 13:22159. 10.1038/s41598-023-49162-6 38092791 PMC10719334

[B122] Zatorre R. J. (2015). Musical pleasure and reward: Mechanisms and dysfunction. *Ann. N. Y. Acad. Sci.* 1337 202–211. 10.1111/nyas.12677 25773636

[B123] Zhang X.-Y. Li J.-J. Lu H.-T. Teng W.-J. Liu S.-H. (2020). Positive effects of music therapist’s selected auditory stimulation on the autonomic nervous system of patients with disorder of consciousness: A randomized controlled trial. *Neural Regen. Res.* 16 1266–1272. 10.4103/1673-5374.301021 33318404 PMC8284264

[B124] Zhou M. Wei C. Xie X. Wang Z. Chen L. Zhang X. (2025). Effect of music therapy on cognitive function among older adults: A systematic review and meta-analysis of randomized controlled trials. *Gerontologist* 65:gnaf106. 10.1093/geront/gnaf106 40084508

